# Viral pancreatitis: research advances and mechanisms

**DOI:** 10.3389/fmicb.2023.1326837

**Published:** 2024-02-14

**Authors:** Xianqiang Yu, Minchao Wang, Qingming Kong

**Affiliations:** ^1^Medical School, Qingdao University, Qingdao, China; ^2^Lishui Second People’s Hospital Affiliated to Wenzhou Medical University, Lishui, China; ^3^School of Basic Medicine and Forensics, Key Laboratory of Bio-tech Vaccine of Zhejiang Province, Engineering Research Center of Novel Vaccine of Zhejiang Province, Hangzhou Medical College, Hangzhou, China; ^4^Key Laboratory of Biomarkers and In Vitro Diagnosis Translation of Zhejiang province, School of Laboratory Medicine and Bioengineering, Hangzhou Medical College, Hangzhou, China

**Keywords:** viral pancreatitis, acute pancreatitis, angiotensin-converting enzyme, acinar cells, virus

## Abstract

Acute pancreatitis is caused by trypsinogen activation in acinar cells caused by various injury forms (gallstone, high triglycerides, alcohol, etc.). Viral pancreatitis is a clinically rare disease type, which is easily neglected by clinicians and causes serious adverse consequences. Viral pancreatitis involves the entry of viruses into pancreatic cells, triggering inflammation, immune response activation, and enzymatic autodigestion, leading to tissue damage and potential complications. At present, there are few available reports on viral pancreatitis, most of which are case reports. This review brings attention to clinicians by describing the incidence of viral pancreatitis to enhance clinical understanding and patient care.

## Introduction

Acute pancreatitis (AP) is a common acute abdominal disease, and its incidence may vary based on geographical, cultural, and demographic factors. For example, AP is the number one cause of acute hospitalizations of the digestive system in the United States (Fagenholz et al., 2007; Peery et al., 2012). Various forms of injury (gallstone, hypertriglyceride, alcohol, etc.) induce the activation of trypsin in acinus cells, which in turn induces acute pancreatitis (; Crockett et al., 2018). Gallstones are a common trigger for AP. The pancreas and gallbladder share a drainage system through the common bile duct (Garcia et al., 2019; Moody et al., 2019). If a gallstone migrates and obstructs this duct, it can lead to a backup of digestive enzymes within the pancreas, causing inflammation. Other factors can also contribute to AP. For example, chronic and excessive alcohol intake can trigger the release of pancreatic enzymes prematurely within the pancreas, causing inflammation (Maleth et al., 2015). Hypertriglyceridemic pancreatitis (HTGP) is a specific subtype of AP triggered by elevated levels of triglycerides in the blood (Carr et al., 2016; Adiamah et al., 2018). Triglycerides are a type of fat that the body uses for energy storage. When levels become excessively high, particularly above 1,000 mg/dL, they can lead to the development of HTGP. In summary, the etiology of AP involves various factors that can initiate a cascade of events leading to pancreatic injury, inflammation, and potentially systemic complications. The overall clinical mortality rate was close to 10%, and over 30% were from severe necrotizing pancreatitis (IAP/APA evidence-based guidelines for the management of acute pancreatitis, 2013).

Viral infections, bacterial infections and parasitic infections can also trigger pancreatitis by direct invasion, immune response, or obstruction of pancreatic ducts. However, acute pancreatitis directly induced by viral infection, which is relatively rare in clinical practice, is easy to be ignored or confused. Certain viruses can directly or indirectly contribute to the development of pancreatitis. The pancreas is a sensitive organ, and viral infections can impact its structure and function. At present, most of the available studies on viral pancreatitis are case reports, so reliable clinical research is necessary to explore the pathogenesis characteristics of viral pancreatitis. Especially during COVID-19 outbreaks, the risk of viral pancreatitis should be considered and clarified.

## Diagnostic criteria

The diagnosis of acute pancreatitis is established by referring to the Revised Atlanta Classification (RAC), and its severity is classified into mild, moderately severe, and severe (Tenner et al., 2013). Acute pancreatitis can be diagnosed if at least two of the following three criteria are fulfilled: abdominal pain (acute onset of persistent and severe epigastric pain, often radiating to the back); serum lipase (or amylase) activity at least three times the upper limit of normal; or characteristic findings of acute pancreatitis on contrast-enhanced CT or, less often, MRI or transabdominal ultrasonography. The diagnosis of viral pancreatitis is mainly based on the discovery of virus in pancreas tissues at autopsy or on biopsy and the exclusion of acute pancreatitis caused by any other etiology. It is important to note that viral pancreatitis cannot be confirmed by laboratory etiology alone.

## Viral pathogen

At present, the pathogen of known viral pancreatitis mainly includes mumps virus, epstein–barr virus, hepatitis virus, coxsackie virus, echoviruses, cytomegalovirus, epidemic hemorrhagic fever virus, measles virus, human immunodeficiency virus and so on (Table 1) (Simons-Linares et al., 2021). In addition, as the COVID-19 pandemic continues, more and more evidence shows that novel coronavirus can directly induce acute pancreatitis (Alves et al., 2020; Gupta et al., 2020; Inamdar et al., 2020; Karimzadeh et al., 2020). We hereby name it COVID-19 associated pancreatitis.

**Table 1:** Characteristics of viral pancreatitis reported in the literature.

Virus type	proportion
Viral hepatitis (A, B, C, D, E)	34.4%
Coxsackie viruses & echoviruses	14.8%
Hemorrhagic fever viruses	12.4%
Cytomegalovirus	12.0%
Varicella zoster virus	10.5%
Measles virus	3.8%
Human immunodeficiency virus	3.8%
Others	8.3%
Severity	
Mild	43.1%
Moderate	11.7%
Mild to moderate	12.8%
Severe	32.4%
Complications	
Pancreatic pseudocysts	7.6%
Necrosis	17.4%
Death	20.1%

## Mechanism of viral associated pancreatitis

**Figure 1:**
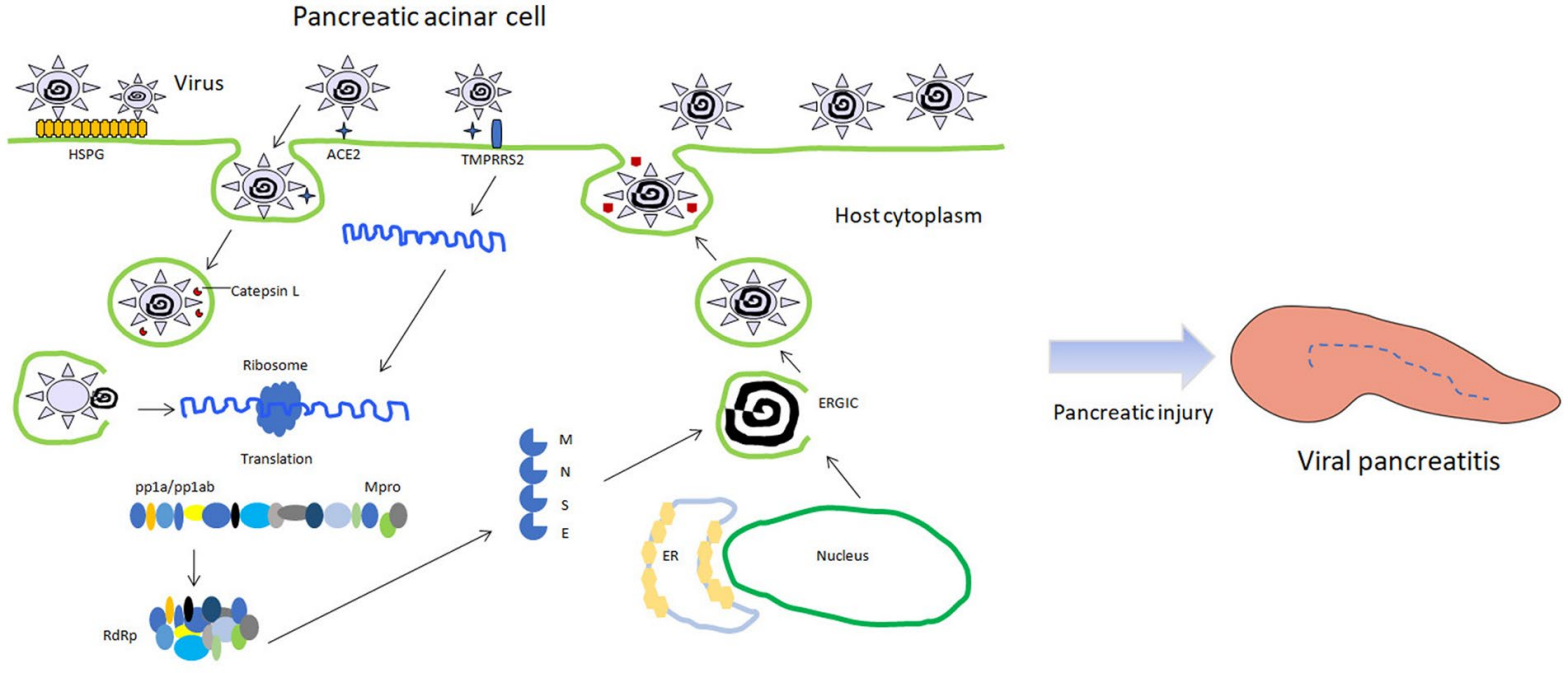
Schematic diagram of viral pancreatitis induced by viral infection of pancreatic acinar cells. HSPG: heparin sulfate proteogylican; TMPRSS2: transmembrane protease serine 2; RdRp: RNA dependent RNA polymerase; Mpro: main protease; ERGIC: endoplasmic reticulum-golgi intermediate compartment; pp1a/pp1ab: polymeric protein 1a/1ab.

Mechanisms of cellular injury: When the virus infects acinar cells, it mediates the transfer of the virus into the cells through the action of angiotensin-converting enzyme 2 (ACE2), thereby initiating further damage (Figure 1) (Lakshmanan and Malik, 2020). The triggering mechanism of pancreatitis: When the virus successfully enters the acinar cells, it prevents the cells from playing the role of exocytosis in a certain way, thus forcing the zymogen granules (containing digestive enzymes) to remain in the cell. The zymogen granules combine with intracellular lysosomes in acinar cells to form condensing or autophagic vacuoles containing an admixture of digestive and lysosomal enzymes. The lysosomal enzyme cathepsin B can activate the conversion of trypsinogen to trypsin. Finally, the accumulated intracellular trypsin exerts its own digestive damage through cascade action (Gaisano et al., 2001; Gukovsky et al., 2012; Sendler et al., 2018). This is similar to the process of virus entering acinar cells in the 2003 outbreak, and ACE2 receptor may be the key factor mediating the damage of most virus-infected acinar cells. In addition, inflammatory cells play a significant role in the development and progression of viral pancreatitis (Aghdassi et al., 2018). When a viral infection occurs in the pancreas, immune cells, such as macrophages, dendritic cells, and T lymphocytes, are recruited to the site (Armstrong et al., 2013). These immune cells recognize viral particles and initiate an immune response to neutralize and eliminate the virus. Inflammatory cells release various chemical messengers called cytokines. Cytokines help orchestrate the immune response by activating other immune cells, promoting inflammation, and signaling for tissue repair. However, an excessive release of cytokines can lead to an exaggerated and damaging inflammatory response. Inflammatory cells infiltrate the pancreatic tissue, contributing to the characteristic swelling, redness, and heat associated with inflammation. What’s more, activated immune cells generate reactive oxygen species (ROS) as part of the immune response (Wang et al., 2015; Sarshari et al., 2023). While ROS play a role in eliminating pathogens, excessive ROS production can lead to oxidative stress, damaging cellular components and contributing to tissue injury. The immune response, along with viral infection and other factors, can lead to cellular damage and death. This can result in the release of intracellular contents and further aggravate inflammation, potentially leading to the development of necrotic tissue and even organ failure.

Viral pancreatitis is a relatively uncommon manifestation of viral infections, but several viruses have been associated with pancreatitis (Hatch-Vallier et al., 2021; Almutairi et al., 2022; Choden et al., 2023). Viruses may have unique ways of interacting with host cells, so the specific mechanisms of pancreatitis may vary. Some viruses may directly infect pancreatic cells, while others may induce an inflammatory response or trigger immune-mediated damage. The severity and clinical presentation of viral pancreatitis can also differ depending on the underlying virus. It is still uncertain whether all the occurrence of viral pancreatitis is caused by ACE2-mediated cell injury. However, viruses may indeed employ different mechanisms that may lead to viral pancreatitis. Virus may interact with pancreatic cells in distinct ways, resulting in inflammation and damage to the pancreas. For example, Mumps is a well-known cause of viral pancreatitis, especially in children. The virus can directly infect and damage pancreatic cells, leading to inflammation (Simons-Linares et al., 2021). Mumps-related pancreatitis is often a part of systemic involvement in mumps infection. However, the way coronavirus invades cells provides reference and possibility for us to explore virus damage. However, in an *in vitro* model of acute pancreatitis, ACE2-angiotensin-(1–7)-Mas axis has been observed to protect against AP (Wang et al., 2015). From this point of view, what specific role does ACE2 play in the development and progression of acute pancreatitis still needs further study.

However, the immunocompromised status of patients can indeed play a role in the development and severity of viral-induced acute pancreatitis. Immunocompromised individuals, such as those with weakened immune systems due to conditions like varicella zoster virus/cytomegalovirus, organ transplantation, or immunosuppressive therapy, may be more susceptible to viral infections (Oku et al., 2005; Picod et al., 2017; Ahmad et al., 2021). Viruses that can cause pancreatitis may take advantage of the compromised immune response, leading to more severe and prolonged episodes of inflammation in the pancreas. It’s important to note that the relationship between viral infections, immunocompromised status, and acute pancreatitis is complex, and not all cases of viral-induced pancreatitis occur in immunocompromised individuals. The specific viruses involved and the mechanisms by which they induce pancreatitis can vary.

## Clinical characteristics

Pancreatitis induced by different types of hepatitis virus infection accounted for the largest proportion of reported cases, about one third. Coxsackie and echoviruses come next. Abdominal pain is the predominant clinical manifestation of almost all viral pancreatitis and is accompanied by an increase in amylase or lipase. At present, the unique clinical manifestations of viral pancreatitis have not been reported (Simons-Linares et al., 2021). It should also be emphasized that some reported diagnoses of viral pancreatitis are not based on isolation of the virus from pancreatic tissue. It’s just laboratory virus testing on the basis of excluding other causes. This means that these cases may be false-positive or not really viral pancreatitis. Discussing the specific signs and symptoms associated with viral-induced pancreatitis can aid clinicians in prompt diagnosis and intervention. The main complications associated with viral pancreatitis include pancreatic necrosis, pseudocysts, abscess formation, organ failure, sepsis, and in severe cases, the development of chronic pancreatitis. These complications underscore the importance of timely diagnosis and appropriate management to prevent adverse outcomes in individuals with viral pancreatitis.

## Therapy and control

Antiviral therapy should be a necessary treatment for viral pancreatitis, even if it does not eradicate the virus. The following treatment of viral pancreatitis should refer to the latest version of clinical treatment guidelines-- revision of the Atlanta classification (RAC), and select individualized treatment plans, including medication, fluid resuscitation, nutritional support, antibiotic use, pain management, complication management, and etiology management, etc. (Tenner et al., 2013). In particular, virus-specific treatment is needed. In addition, ACE2 may provide a new target for the treatment of viral pancreatitis in clinical practice (Yamaya et al., 2020).

Most importantly, once viral pancreatitis is confirmed, quarantine measures and relevant protective measures should be taken as soon as possible to prevent potential viral transmission. Clinicians should explore the characteristics of the virus and the source of infection, and identify the primary organs of virus infection, so as to make timely clinical decisions. In addition, it is equally important to explore preventive measures, such as vaccination strategies against viruses such as mumps. Highlighting the distinctive therapeutic considerations for viral pancreatitis ensures a more nuanced approach to patient management.

In addition, clinicians should distinguish between the following two concepts: acute pancreatitis combined with virus infection and viral pancreatitis. This is necessary because the treatment of patients in the context of the pandemic requires such consideration. Acute pancreatitis combined with virus infection means that the occurrence of the two diseases overlaps in time, but is not cause-and-effect. However, viral pancreatitis indicates that acute pancreatitis is caused by viral infection. It is more convenient for the clinician to make appropriate clinical judgment to distinguish the difference between them.

## Conclusion

Viral pancreatitis refers to a specific type of AP that is triggered or influenced by viral infections. It is a rare etiological type in clinical practice, and it is very easy to be missed or misdiagnosed by clinicians, which not only delays the patient’s condition, but also causes the risk of the spread of potential viral infections. Viral pancreatitis can present with symptoms similar to other causes of AP, including severe abdominal pain, nausea, vomiting, and elevated pancreatic enzymes. Diagnosis and management should be conducted by qualified medical professionals. Always refer to authoritative medical sources for accurate and up-to-date information on viral pancreatitis and its associated viruses. Especially in the context of the current COVID-19 pandemic, there is indeed a need for adequate vigilance and attention.

## Author contributions

XY: Methodology, Investigation, Formal analysis, Writing – original draft. MW: Writing – original draft. QK: Project administration, Writing – review & editing, Funding acquisition.
